# Rates of Diabetes-Related Major Amputations Among Racial and Ethnic Minority Adults Following Medicaid Expansion Under the Patient Protection and Affordable Care Act

**DOI:** 10.1001/jamanetworkopen.2022.3991

**Published:** 2022-03-24

**Authors:** Tze-Woei Tan, Elizabeth A. Calhoun, Shannon M. Knapp, Adelina I. Lane, David G. Marrero, C. Kent Kwoh, Wei Zhou, David G. Armstrong

**Affiliations:** 1 Department of Surgery, University of Arizona College of Medicine, Tucson; 2 Southwestern Academic Limb Salvage Alliance (SALSA), Los Angeles, California; 3 Department of Population Health, University of Kansas Medical Center, Kansas City; 4 Statistics Consulting Lab, Bio5 Institute, University of Arizona, Tucson; 5 Center for Border Health Disparities, University of Arizona Health Science, Tucson; 6 Department of Medicine, University of Arizona College of Medicine, Tucson; 7 Department of Surgery, Keck School of Medicine of University of Southern California, Los Angeles

## Abstract

**Importance:**

It is not known whether implementation of Medicaid expansion under the Patient Protection and Affordable Care Act (ACA) was associated with improvements in the outcomes among racial and ethnic minority adults at risk of diabetes-related major amputations.

**Objective:**

To explore the association of early Medicaid expansion with outcomes of diabetic foot ulcerations (DFUs).

**Design, Setting, and Participants:**

This cohort study included hospitalizations for DFUs among African American, Asian and Pacific Islander, American Indian or Alaska Native, and Hispanic adults as well as adults with another minority racial or ethnic identification aged 20 to 64 years. Data were collected from the State Inpatient Databases for 19 states and the District of Columbia for 2013 to the third quarter of 2015. The analysis was performed on December 4, 2019, and updated on November 9, 2021.

**Exposures:**

States were categorized into early-adopter states (expansion by January 2014) and nonadopter states.

**Main Outcomes and Measures:**

Poisson regression was performed to examine the associations of state type, time, and their combined association with the proportional changes of major amputation rate per year per 100 000 population.

**Results:**

Among the 115 071 hospitalizations among racial and ethnic minority adults with DFUs (64% of sample aged 50 to 64 years; 35%, female; 61%, African American; 25%, Hispanic; 14%, other racial and ethnic minority group), there were 36 829 hospitalizations (32%) for Medicaid beneficiaries and 10 500 hospitalizations (9%) for uninsured patients. Hospitalizations increased 3% (95% CI, 1% to 5%) in early-adopter states and increased 8% (95% CI, 6% to 10%) in nonadopter states after expansion, a significant difference (*P *for interaction < .001). Although there was no change in the amputation rate (0.08%; 95% CI, −6% to 7%) in early-adopter states after expansion, there was a 9% (95% CI, 3% to 16%) increase in nonadopter states, a significant change (*P* = .04). For uninsured adults, the amputation rate decreased 33% (95% CI, 10% to 50%) in early-adopter states and did not change (12%; 95% CI, −10% to 38%) in nonadopter states after expansion, a significant difference (*P* = .006). There was no difference in the change of amputation rate among Medicaid beneficiaries between state types after expansion.

**Conclusions and Relevance:**

This study found a relative improvement in the major amputation rate among African American, Hispanic, and other racial and ethnic minority adults in early-expansion states compared with nonexpansion states, which could be because of the recruitment of at-risk uninsured adults into the Medicaid program during the first 2 years of ACA implementation. Future study is required to evaluate the long-term association of Medicaid expansion and the rates of amputation.

## Key Points

**Question:**

Has the amputation rate among African American, Hispanic, and other racial and ethnic minority adults with diabetic foot ulcerations (DFUs) changed during the early implementation of the Patient Protection and Affordable Care Act (ACA)?

**Findings:**

In a cohort study of 115 071 hospitalizations for DFUs among racial and ethnic minority adults, early Medicaid expansion was associated with decreased major amputation and hospitalization rates in early-adoption states compared with nonadoption states.

**Meaning:**

These findings suggest that early ACA implementation was associated with decreased lower extremity amputation among underserved racial and ethnic minority adults with DFUs.

## Teaser

This cohort study explores the association of early Medicaid expansion with outcomes of diabetic foot ulcerations (DFUs).

## Introduction

Racial and ethnic minority adults experience a disproportionately elevated risk of lower extremity amputation.^1,2,3,4,5^ Diabetic foot ulceration (DFU), which precedes more than 80% of amputations, is the leading cause of limb loss in minority individuals with diabetes.^6^ African American, Hispanic, and American Indian individuals with DFUs have a several-fold greater risk of amputation than their White counterparts.^2,5,7^ One of the contributing factors may be a barrier to medical care specifically due to the lack of health insurance.^1^

The Patient Protection and Affordable Care Act (ACA) is a health care reform law enacted in March 2010.^8^ One of the mandates of the ACA, which became effective on January 1, 2014, was to expand the eligibility of Medicaid programs to include nonelderly adults with income up to 138% of the federal poverty level.^8^ In 2012, the Supreme Court ruled to allow individual states to opt-out of the ACA Medicaid expansion.^9^ Following January 2014, 12 states and the District of Columbia expanded, and 14 states did not.^10^ This variation in the implementation presented an opportunity to evaluate the association of ACA with care and outcomes in the racial and ethnic minority population.

By early 2016, a significant number of Hispanic and African American adults gained health insurance coverage due to the ACA.^11,12^ Studies have also found an increase in the number of newly diagnosed diabetes and a reduction in short-term diabetes-related complications.^13,14,15^ However, the association of the early ACA implementation on the outcomes of underserved populations at risk of diabetes-related amputations is unclear. In this study, we hypothesized that African American, Hispanic, and other racial and ethnic minority adults with DFUs in states that expanded Medicaid would have improved outcomes compared with those in nonexpansion states.

## Methods

### Data Sources

State Inpatient Databases (SID) from 2013, 2014, and through the third quarter of 2015 were queried to identify hospitalizations with DFUs. The SID includes 97% of all US hospital discharges from acute care hospitals. The exposure was expansion of Medicaid after ACA implementation (ie, early-adopter states), with states that did not expand Medicaid as the reference group (ie, nonadopter states). Our study cohort included hospitalizations from 12 states that expanded Medicaid by January 1, 2014 (ie, Arizona, Colorado, Iowa, Kentucky, Massachusetts, Maryland, New Jersey, New Mexico, Nebraska, New York, Rhode Island, and Washington) and the District of Columbia and 7 states that did not expand (ie, Florida, Georgia, Kansas, Mississippi, North Carolina, South Carolina, and South Dakota). We did not include 18 expansion states because they enacted expansion after January 1, 2014 (Maine, Michigan, Pennsylvania, and Utah), are not on the US mainland (Alaska and Hawaii), or the data for 2013, 2014, and 2015 were not available in the Healthcare Cost and Utilization Project (HCUP; California, Connecticut, Delaware, Idaho, Illinois, Indiana, Missouri, Montana, North Dakota, New Hampshire, Oklahoma, and Virginia). In addition, 4 nonexpansion states were excluded because of data not being available in the HCUP (Alabama, Tennessee, Texas, and Wyoming).

The institutional review board at the University of Arizona exempted this study for review and the requirement for informed consent because of the use of unidentifiable data. The study followed the Strengthening the Reporting of Observational Studies in Epidemiology (STROBE) reporting guidelines.^16^

### Study Populations

We used the *International Classification of Diseases, Ninth Revision *(*ICD-9*) diagnosis codes to identify the study cohort. Hospitalizations with *ICD-9* codes of diabetes (250.xx) and 1 of the following *ICD-9* codes were included: 707.1x, 707.8, 707.9, 440.23, 440.24, 785.4, 040.0, 730.07, 730.17, 730.27, 730.97, 681.1, or 682.7. We excluded hospitalizations with (1) missing value for race and ethnicity, gender, or insurance and (2) data from the fourth quarter of 2015 because of the transition of *ICD-9* codes to *International Statistical Classification of Diseases and Related Health Problems, Tenth Revision *(*ICD-10*) codes to ensure the consistency.

### Outcomes

Two main outcomes were hospitalizations and major amputations for DFUs. These included both above-knee amputation and below-knee amputation (*ICD-9* procedure codes: 84.10, 84.13-17, and 84.3; *Current Procedure Terminology* [*CPT*] codes: 27590-92, 27594, 27596, 27598, 27880-82, 27884, 27886, 27888, and 27889).

### Variables and Covariates

We identified hospitalizations based on race and ethnicity information, which included White, African American, Hispanic, Asian, American Indian, and other adults. We categorized Asian, American Indian, and other into the same other category because of small numbers.

### Statistical Analysis

The characteristics are described as a frequency and percentage for categorical variables and as mean and SD for continuous variables. For the analysis, the outcomes were identified using the first 6 *ICD-9* procedure codes and the first 11 *CPT* codes. We used the US Census Bureau to obtain the numbers of minority and White adults (aged 20-64 years) from 2013 to 2015 in the included early-adopter states and nonadopter states. The data were stratified by racial and ethnic group and by the type of insurance (based on expected primary payer) to calculate the rate of hospitalization and the rate of major amputation.

For each outcome, we looked at 2 Poisson regression models. We assumed that the early-adopter states and nonadopter states had similar pre-expansion and postexpansion time trends without ACA implementation. Model 1 included hospitalizations regardless of insurance and included effects of state type (ie, early-adopter state vs nonadopter state), time (ie, pre-ACA vs post-ACA), and race and ethnicity. Model 2 included only hospitalizations among Medicaid beneficiaries or uninsured adults and included an effect of insurance in addition to the effects in model 1. Both models included interactions of all terms and an offset for the population size. For pre-ACA counts, the offset was equal to the 2013 population. Because post-ACA counts included events over a 1.75-year time period, the offset was equal to the 2014 population plus 75% of the 2015 population to account for differences in population size and time span; results are presented as estimates of outcome rates, in per population (eg, hospitalizations per 100 000 population), and per year.

Within each group we estimated (point estimate and 95% CI) the proportional change in outcome rate (per year per 100 000) post-ACA vs pre-ACA. The primary test of interest was whether the change differed between state types within a group and was based on an interaction effect. The model is analogous to testing whether the difference in differences is equal to 0, but proportional change is the appropriate measure rather than differences in absolute terms.

Analysis was initially performed on December 4, 2019, and updated on November 9, 2021. The data analyses were performed using SAS software version 9.4 (SAS Institute) and R software version 3.6.3 (R Project for Statistical Computing). The glht function in R package multcomp was used to compute confidence intervals and *P* values for linear combinations of parameters used to estimate the outcomes of interest.^17^ Statistical significance was set at *P* < .05, and all tests were 2-tailed.

## Results

There were 115 071 hospitalizations (73 760 [64%] aged 50 to 64 years; 40 144 [35%] female) for DFUs in African American adults (69 648 [61%]), Hispanic adults (29 264 [25%]), and adults in the other category (16 159 [14%]); 36 829 hospitalizations (32.0%) were among Medicaid beneficiaries and 10 500 (9.1%) for uninsured individuals. There were 152 986 hospitalizations for White adults with DFUs (32 564 [21.3%] Medicaid beneficiaries and 11 562 [7.6%] uninsured). Baseline characteristics for hospitalizations (including all insurance types) with DFUs and major amputation are listed in Table 1 and Table 2.

**Table 1.** Baseline Characteristics and Outcomes for Hospitalizations of Adults With Diabetic Foot Ulcerations in Early-Adopter and Nonadopter States Before and After Medicaid Expansion

Characteristics	Hospitalizations, No. (%)			
	Early-adopter states		Nonadopter states	
	Pre-ACA (n = 52 854)	Post-ACA (n = 100 078)	Pre-ACA (n = 38 880)	Post-ACA (n = 76 239)
Age, y				
20-34	2031 (3.8)	4103 (4.1)	1863 (4.8)	3642 (4.8)
35-49	13 447 (25.4)	24 937 (24.9)	10 452 (26.9)	20 243 (26.6)
50-64	37 376 (70.7)	71 038 (71.0)	26 565 (68.3)	52 354 (68.7)
Gender				
Male	35 693 (67.5)	68 500 (68.4)	25 165 (64.7)	49 464 (64.9)
Female	17 161 (32.5)	31 578 (31.6)	13 715 (31.6)	26 775 (35.1)
Race/ethnicity				
African American	11 029 (20.9)	20 082 (20.1)	13 125 (33.8)	25 412 (33.3)
Hispanic	7048 (13.3)	12 652 (12.6)	3249 (8.4)	6315 (8.3)
White	30 580 (57.9)	58 878 (58.8)	21 360 (54.9)	42 162 (55.3)
Other^a^	4197 (7.9)	8466 (8.5)	1146 (2.9)	2350 (3.1)
Health insurance				
Medicaid	13 222 (25.0)	31 812 (31.8)	8422 (21.7)	15 935 (20.9)
Medicare	18 772 (35.5)	35 447 (35.4)	15 222 (39.2)	29 778 (39.1)
Other	1740 (3.3)	2102 (2.1)	1689 (4.3)	3170 (4.2)
Private	14 687 (27.8)	27 272 (27.3)	8456 (21.7)	18 264 (24.0)
Uninsured	4433 (8.4)	3445 (3.4)	5091 (13.1)	9092 (11.9)
Area of residency				
Not urban	2803 (5.3)	4935 (4.9)	3399 (8.7)	6636 (8.7)
Urban	50 051 (94.7)	95 143 (95.1)	35 481 (91.3)	69 603 (91.3)
Medical comorbidities				
Hypertension	36 316 (68.7)	68 375 (68.3)	28 037 (72.1)	54 540 (71.5)
Peripheral artery disease	12 879 (24.4)	23 680 (23.7)	9116 (23.4)	17 651 (23.2)
Congestive heart failure	6817 (12.9)	13 766 (13.8)	5037 (13.0)	10 457 (13.7)
Chronic lung disease	7613 (14.4)	14 271 (14.3)	5047 (13.0)	10 054 (13.2)
Kidney failure	16 446 (31.1)	30 951 (30.9)	12 490 (32.1)	24 551 (32.2)
Outcomes				
Major amputation	3445 (6.5)	6490 (6.5)	3125 (8.0)	6097 (8.0)
Length of stay, mean (SD), d	7.92 (9.35)	7.92 (9.05)	8.11 (8.64)	8.03 (8.68)

Abbreviation: ACA, the Patient Protection and Affordable Care Act.

^a^ Other includes Asian or Pacific Islander, American Indian or Alaska Native, and Other.

**Table 2.** Baseline Characteristics and Outcomes for Hospitalizations of Adults With a Major Amputation in Early-Adopter and Nonadopter States Before and After Medicaid Expansion^a^

Characteristics	No. (%)			
	Early-adopter states		Nonadopter states	
	Pre-ACA (n = 3445)	Post-ACA (n = 6490)	Pre-ACA (n = 3125)	Post-ACA (n = 6097)
Age, y				
20-34	<5%	<5%	<5%	<5%
35-49	776 (22.5)	1375 (21.2)	681 (21.8)	1327 (21.8)
50-64	2600 (75.5)	4980 (76.7)	2371 (75.9)	4630 (75.9)
Gender				
Male	2440 (70.8)	4678 (72.1)	2140 (68.5)	4087 (67.0)
Female	1005 (29.2)	1812 (27.9)	985 (31.5)	2010 (33.0)
Race and ethnicity				
African American	819 (23.8)	1428 (22.0)	1365 (43.7)	2717 (44.6)
Hispanic	452 (13.1)	833 (12.8)	186 (6.0)	337 (5.5)
White	1910 (55.4)	3728 (57.4)	1490 (47.7)	2868 (47.0)
Other^b^	264 (7.7)	501 (7.7)	<5%	<5%
Health insurance				
Medicaid	853 (24.8)	1993 (30.7)	708 (22.7)	1343 (22.0)
Medicare	1413 (41.0)	2627 (40.5)	1432 (45.8)	2849 (46.7)
Other	<5%	<5%	<5%	<5%
Private	830 (24.1)	1570 (24.2)	614 (19.6)	1244 (20.4)
Uninsured	240 (7.0)	<5%	269 (8.6)	435 (7.1)
Area of residency				
Not urban	215 (6.2)	370 (5.7)	367 (11.7)	637 (10.4)
Urban	3230 (93.8)	6120 (94.3)	2758 (88.3)	5460 (89.6)
Medical comorbidities				
Hypertension	2325 (67.5)	4359 (67.2)	2313 (74.0)	4450 (73.0)
Peripheral artery disease	1989 (57.7)	3590 (55.3)	1667 (53.3)	3261 (53.5)
Congestive heart failure	622 (18.1)	1199 (18.5)	589 (18.8)	1154 (18.9)
Chronic lung disease	363 (10.5)	701 (10.8)	343 (11.0)	648 (10.6)
Kidney failure	1392 (40.4)	2598 (40.0)	1384 (44.3)	2614 (42.9)
Outcomes				
Length of stay, mean (SD), d	13.71 (12.63)	13.98 (13.85)	12.85 (11.90)	12.88 (11.49)

Abbreviation: ACA, the Patient Protection and Affordable Care Act.

^a^ Cells with small numbers are presented as less than 5% per the requirements for publishing Healthcare Cost and Utilization Project data.

^b^ Other includes Asian or Pacific Islander, American Indian or Alaska Native, and other.

### Rate of Major Amputation per 100 000 per Year for Adults, Including All Insurance Types

**Figure 1.**
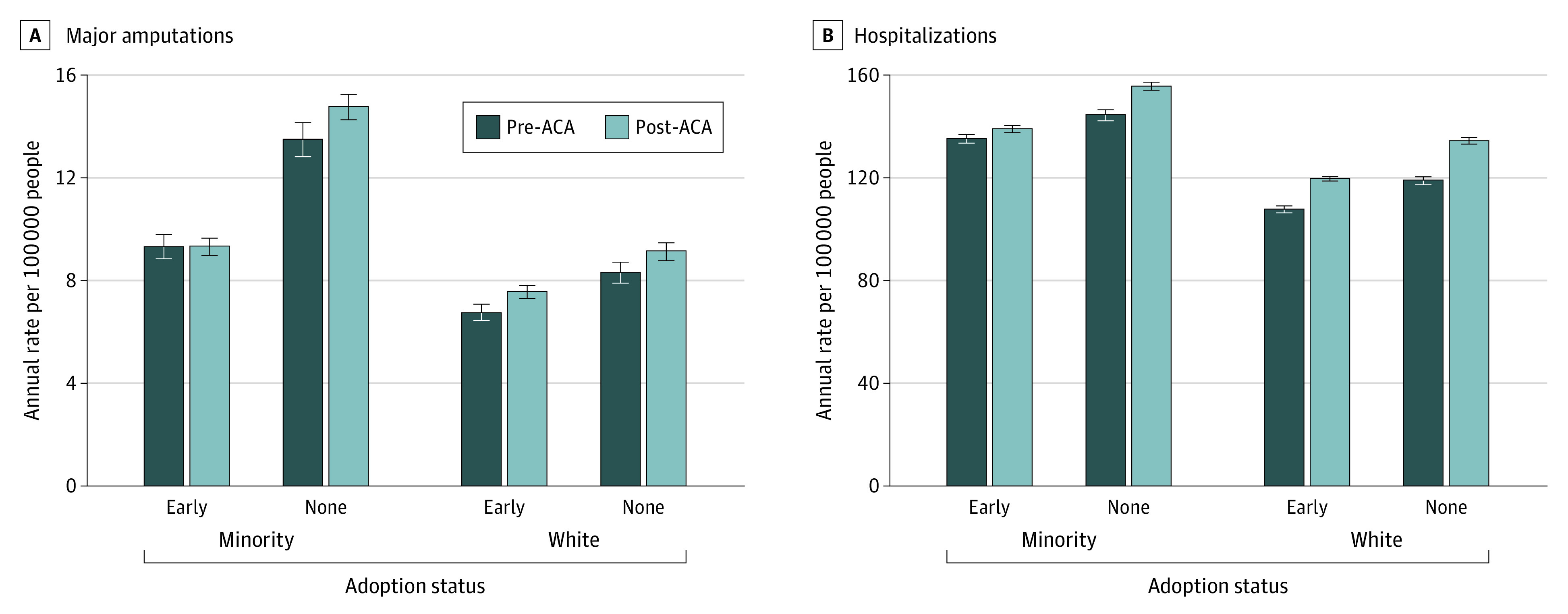
Rate of Major Amputation and Hospitalizations for Adults With All Insurance Types With Diabetic Foot Ulcerations Before and After Medicaid Expansion in Early-Adopter and Nonadopter States Minority group includes African American adults, Hispanic adults, and adults with another racial or ethnic minority identification. ACA indicates the Patient Protection and Affordable Care Act.

There was no significant change in the rate of amputation among African American adults, Hispanic adults, and adults in the other category (estimated change: 0.08%; 95% CI, −6% to 7%) in early-adopter states (from 9.3 per 100 000 population per year to 9.3 per 100 000 population per year) after expansion. In nonadopter states, the rate of amputation increased 9% (95% CI, 3% to 16%; from 13.5 per 100 000 population per year to 14.8 per 100 000 population per year) after expansion. The relative difference in the changes between state types was significant (*P* = .04) for racial and ethnic minority adults (Table 3 and Figure 1).

**Table 3.** Proportional Change in the Rate of Major Amputation and Rate of Hospitalization in Early-Adopter and Nonadopter States for Adults With Diabetic Foot Ulcerations After Medicaid Expansion

Group	Estimate (95% CI)	*P* value	*P *for interaction
**Hospitalization**			
All insurance types			
Racial and ethnic minority^a^			
Early-adopter	1.03 (1.01-1.05)	<.001	<.001
Nonadopter	1.08 (1.06-1.10)	<.001	
White			
Early-adopter	1.11 (1.09-1.12)	<.001	.09
Nonadopter	1.13 (1.11-1.15)	<.001	
Medicaid beneficiaries and uninsured patients			
Racial and ethnic minority Medicaid^a^			
Early-adopter	1.05 (1.02-1.08)	<.001	<.001
Nonadopter	0.96 (0.93-1.00)	.03	
Racial and ethnic minority uninsured^a^			
Early-adopter	0.65 (0.61-0.69)	<.001	<.001
Nonadopter	1.26 (1.20-1.33)	<.001	
White Medicaid			
Early-adopter	1.09 (1.06-1.12)	<.001	<.001
Nonadopter	0.99 (0.96-1.03)	.76	
White uninsured			
Early-adopter	0.61 (0.57-0.65)	<.001	<.001
Nonadopter	1.27 (1.21-1.33)	<.001	
**Major amputation**			
All insurance			
Racial and ethnic minority^a^			
Early-adopter	1.00 (0.94-1.07)	.98	.04
Nonadopter	1.09 (1.03-1.16)	.003	
White			
Early-adopter	1.12 (1.06-1.19)	<.001	.63
Nonadopter	1.10 (1.04-1.17)	.003	
Medicaid beneficiaries and uninsured patients			
Racial and ethnic minority Medicaid^a^			
Early-adopter	0.98 (0.88-1.09)	.66	.98
Non-adopter	0.97 (0.86-1.10)	.65	
Racial and ethnic minority uninsured^a^			
Early-adopter	0.67 (0.50-0.90)	.008	.006
Nonadopter	1.12 (0.9-1.38)	.31	
Interaction			
White Medicaid			
Early-adopter	1.11 (0.99-1.25)	.08	.20
Nonadopter	0.99 (0.86-1.14)	.85	
White uninsured			
Early-adopter	0.57 (0.44-0.73)	<.001	<.001
Nonadopter	1.18 (0.95-1.46)	.14	

^a^ Racial and ethnic minority includes African American adults, Hispanic adults, and adults with other minority racial and ethnic group identification.

The rate of major amputation among White adults increased 12% (95% CI, 6%-19%) in early-adopter states (from 6.7 per 100 000 population per year to 7.6 per 100 000 population per year) and increased 10% (95% CI, 3%-17%) in nonadopter states (from 8.3 per 100 000 population per year to 9.2 per 100 000 population per year) after expansion. The relative difference in the changes between state types for White adults was not significant (*P* = .63) (Table 3 and Figure 1).

### Rate of Hospitalization per 100 000 per Year for Adults, Including All Insurance Types

The rate of hospitalization among African American adults, Hispanic adults, and adults in the other category increased 3% (95% CI, 1%-5%) in early-adopter states (from 135.2 per 100 000 population per year to 139.1 per 100 000 population per year) and increased 8% (95% CI, 6%-10%) in nonadopter states (from 144.5 per 100 000 population per year to 155.7 per 100 000 population per year) after expansion. The relative difference in the changes between state types for racial and ethnic minority adults was statistically significant (*P* < .001) (Table 3 and Figure 1).

For White adults in early-adopter states, the rate of hospitalization increased 11% (95% CI, 9%-12%) from 108.0 per 100 000 population per year to 119.7 per 100 000 population per year after expansion. For White adults in nonadopter states, the rate of hospitalization increased 13% (95% CI, 11%-15%) from 119.1 per 100 000 population per year to 134.5 per 100 000 population per year after expansion. There was no difference (*P* = .09) in the changes between state types for White adults (Table 3 and Figure 1).

### Rate of Major Amputation per 100 000 per Year for Racial and Ethnic Minority Adults With Medicaid or No Insurance

**Figure 2.**
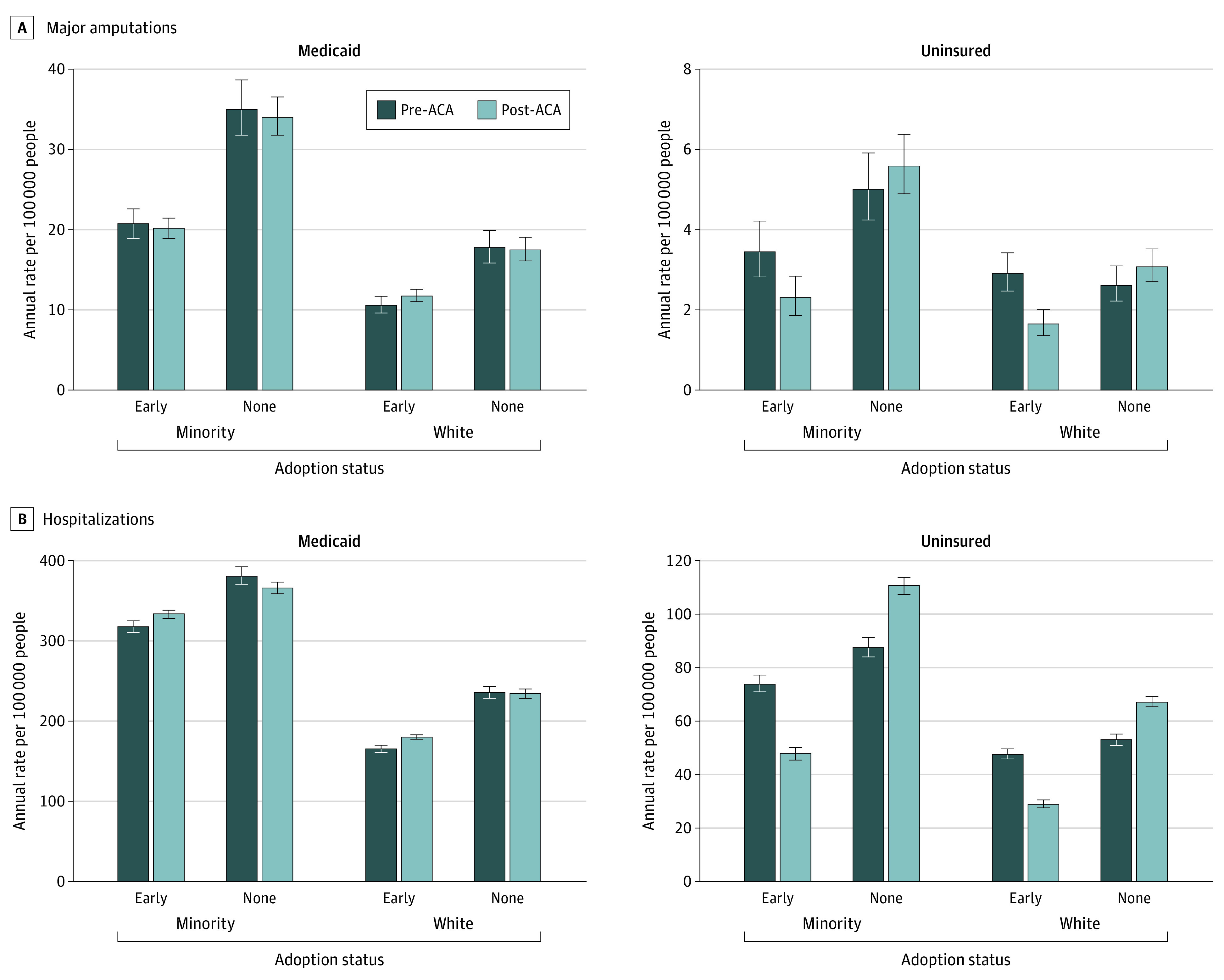
Rate of Major Amputation and Hospitalizations for Medicaid Beneficiaries and Uninsured Adults With Diabetic Foot Ulcerations Before and After Medicaid Expansion in Early-Adopter and Nonadopter States Minority group includes African American adults, Hispanic adults, and adults with another racial or ethnic minority identification. ACA indicates the Patient Protection and Affordable Care Act.

There was no significant difference in the changes of the rate of amputation among racial and ethnic minority adults who were Medicaid beneficiaries in early-adopter states (estimated change: −2%; 95% CI, −12% to 9%; from 20.6 per 100 000 population per year to 20.1 per 100 000 population per year) compared with nonadopter states (estimated change: −3%; 95% CI, −14% to 10%; from 34.9 per 100 000 population per year to 34.0 per 100 000 population per year) after expansion (*P* = .97) (Table 3 and Figure 2).

The rate of amputation among African American adults, Hispanic adults, and adults in the other category without insurance decreased 33% (95% CI, 10% to 50%) in early-adopter states (from 3.4 per 100 000 population per year to 2.3 per 100 000 population per year) and did not change (estimated change: 12%; 95% CI, −10% to 38%) in nonadopter states (5.0 per 100 000 population per year to 5.6 per 100 000 population per year) after expansion. The relative difference in the changes between state types was significant (*P* = .006) for uninsured racial and ethnic minority adults (Table 3 and Figure 2).

### Rate of Hospitalization per 100 000 per Year for Racial and Ethnic Minority Adults With Medicaid or No Insurance

The rate of hospitalization for African American adults, Hispanic adults, and adults in the other category who were Medicaid beneficiaries in early-adopter states was 317.1 per 100 000 population per year before expansion and 332.9 after expansion per 100 000 population per year (estimated change: −5%; 95% CI, −2% to −8%). For African American adults, Hispanic adults, and adults in the other category who were Medicaid beneficiaries in nonadopter states, the rate of hospitalization was 379.7 per 100 000 population per year before expansion and 364.8 per 100 000 population per year after expansion (estimated change: −4%; 95% CI, −7% to −0.004%). The relative difference in the changes between state types was significant (*P* < .001) for racial and ethnic minority Medicaid beneficiaries (Table 3, Figure 2).

For African American adults, Hispanic adults, and adults in the other category without insurance in early-adopter states, the rate of hospitalization decreased 35% (95% CI, 31%-39%) (from 74.1 per 100 000 population per year to 47.8 per 100 000 population per year after expansion). During the same period in nonadopter states, the rate of hospitalization increased 26% (95% CI, 20%-33%) (from 87.8 per 100 000 population per year to 110.8 per 100 000 population per year after expansion) for African American adults, Hispanic adults, and adults in the other category. The relative difference in the changes between state types was significant (*P* < .001) for racial and ethnic minority adults without insurance (Table 3 and Figure 2).

### Rate of Major Amputation per 100 000 per Year for White Medicaid Beneficiaries and Uninsured White Adults

There was no significant difference in the changes between state types for the rate of amputation after expansion among White Medicaid beneficiaries in early-adopter states (estimated change: 11%; 95% CI, −1% to 25%; from 10.6 per 100 000 population per year to 11.7 per 100 000 population per year) compared with non-ACA adopter states (estimated change, −1%; 95% CI, −14% to 14%; 17.7 per 100 000 population per year to 17.5 per 100 000 population per year) after expansion (*P* = .20) (Table 3 and Figure 2).

For uninsured White adults, there was a 43% decrease in the rate of amputation in early-adopter states (95% CI, 27% to 56%; from 2.9 per 100 000 population per year to 1.6 per 100 000 population per year) and no significant change in nonadopter states (estimated change: 18%; 95% CI, −5% to 46%; from 2.6 per 100 000 population per year to 3.1 per 100 000 population per year) after expansion. The relative difference in the changes was significant between state types for uninsured White adults (*P* < .001) (Table 3 and Figure 2).

### Rate of Hospitalization per 100 000 per Year for White Medicaid Beneficiaries and the Uninsured White Adults

The rate of hospitalization for White Medicaid beneficiaries in early-adopter states increased 9% (95% CI, 6% to 12%) from 165.4 per 100 000 population per year before expansion to 180.4 per 100 000 population per year after expansion. For White Medicaid beneficiaries in nonadopter states, the rate of hospitalization was 235.3 per 100 000 population per year before expansion and 233.9 per 100 000 population per year after expansion in nonadopter states (estimated change: −1%; 95% CI, −4% to 3%). The relative changes between state types was significant for White Medicaid beneficiaries (*P* < .001) (Table 3 and Figure 2).

For uninsured White adults in early-adopter states, the rate of hospitalization was 47.7 per 100 000 population per year before expansion and 29.0 per 100 000 population per year after expansion (estimated change: −39%; 95% CI, −43% to −35%). The rate of hospitalization for the uninsured White adults in nonadopter states increased 27% (95% CI, 21% to 33%) from 53.0 per 100 000 population per year before expansion to 67.3 per 100 000 population per year after expansion. The relative changes between state types was significant (*P* < .001) for uninsured White adults (Table 3 and Figure 2).

## Discussion

Our study found that in the first 21 months following Medicaid expansion, the major amputation rate increased 9% among African American adults, Hispanic adults, and adults in the other category in nonadopter states, whereas there was no significant change in early-adopter states. The blunted rise in amputation corresponded with a 33% reduction in major amputation among uninsured African American adults, Hispanic adults, and adults in the other category in early-adopter states after expansion. In comparison, there was no significant change in major amputation rates among uninsured African American adults, Hispanic adults, and adults in the other category in nonadopter states during the same period. Thus, the relative improvement of major amputation rate for DFUs in states that expanded Medicaid suggests a redistribution of at-risk uninsured African American adults, Hispanic adults, and adults in the other category into the Medicaid program. Of note, there was no significant change in the amputation rate for African American adults, Hispanic adults, and adults in the other category who were Medicaid beneficiaries in early-adopter states after expansion.

African American, Hispanic, American Indian adults continue to experience disparities in rates of amputation related to DFUs.^1,2,3,4^ Compared with White adults, a recent study by our group^5^ reported a 33%, 44%, and 47% higher risk for Hispanic, African American, and American Indian individuals with diabetic foot problems, respectively.^5^ Others have similarly found an independent association of race and ethnicity with the risks of amputation.^4,7^ African American, Hispanic, and American Indian adults with diabetes are more likely to have a foot infection and more extensive comorbidities on presentation compared with White adults.^5,6,7,18^ In addition to having a more advanced presentation, the disparities in socioeconomic class and insurance status reflect barriers for accessing medical care. As such, insurance coverage may be responsible for the disparate risks of amputation across the racial and ethnic groups with DFUs.^19,20,21^ Indeed, patients with no health insurance or who are underinsured are more likely to experience diabetes-related complications and lower extremity amputations.^7,22,23^

Hispanic and African American adults had the highest uninsured rates compared to White adults before the ACA.^12,24^ Although disparities in uninsured rates persist, coverage improved substantially across different racial and ethnicity groups after ACA implementation, with the most substantial gain among Hispanic and African American individuals.^12,25^ The improvement in health insurance coverage after the ACA was associated with an increase in the proportion of Medicaid beneficiaries with newly diagnosed diabetes and the number of Medicaid-insured visits for African American and Hispanic individuals with diabetes.^13,14,26^ Our study similarly observed a 35% decrease in hospitalizations for African American adults, Hispanic adults, and adults in the other category without insurance and 5% increase in hospitalizations for Medicaid beneficiaries in expanding states following ACA implementation. In comparison, there was an 26% increase in the hospitalizations among African American adults, Hispanic adults, and adults in the other category in nonadopter states before and after January 2014.

We speculate that the increase in hospitalizations among this group of Medicaid beneficiaries and the reduction in hospitalizations among uninsured racial and ethnic minority adults in early-expansion states in the study could be because of shifting at-risk, uninsured adults into Medicaid. Collectively, the relative improvement in the amputation rate for African American adults, Hispanic adults, and adults in the other category, the decrease in amputation rates among African American adults, Hispanic adults, and adults in the other category without insurance, and the lack of significant change in amputation rate for racial and ethnic minority Medicaid beneficiaries suggest an overall improvement in access to limb salvage care in early-adopter states vs nonadopter states. In comparison, there was no significant change in major amputation among African American adults, Hispanic adults, and adults in the other category without insurance with DFUs in nonadopter states.

A recent study (which included 22 335 individuals with diagnosed diabetes)^15^ reported a significant benefit in self-reported access to health care, self-reported diabetes management, and self-reported health status in Medicaid expansion states compared with states that did not expand, particularly in states with high diabetes rates. Others have also found the association of ACA Medicaid expansion with increased rates of diabetes diagnoses and use of diabetes medications among low-income US residents.^14,27,28^ The reported reduction in potentially avoidable hospitalization for ambulatory care–sensitive conditions after Medicaid expansion, such as short-term diabetes complications, may suggest increased access to ambulatory care.^29^

Few studies have evaluated the association of Medicaid expansion with the rates of diabetes-related amputations. A study by Duke et al^30^ found a decrease in the uninsured population and a 26% reduction in the odds ratio for amputation (*P* < .005) before and after Medicaid expansion in Arkansas.^30^ In a different cohort study of patients with peripheral artery disease,^31^ the disproportionately higher probability of amputation among racial and ethnic minority adults (probability: 8.5%; 95% CI, 3.4%-13.5%) decreased significantly after Medicaid expansion in Massachusetts (probability: 3.9%; 95% CI, 1.3%-9.1%) compared with White patients.

### Limitations

There are several important limitations to this study. First, this is a retrospective study using administrative data and is limited by the use of *ICD-9* diagnosis codes to identify the study cohort. Second, there is a lack of information on the severity of the presentation, including the size of the DFU, the presence of infection, and the extent of peripheral artery disease. Third, we do not have information on the care of DFUs in ambulatory settings before and after hospitalizations. Fourth, we did not include all US states and could not adjust for how the states implemented Medicaid expansion under the ACA; some of the states expanded their Medicaid program under waivers, including those related to work eligibility. Without including all US states, our study sample could lead to representative biases. Fifth, a longitudinal study with a longer study period would be required to assess the durability of ACA Medicaid expansion and the risks of major amputation with DFUs across the racial and ethnic groups.

## Conclusions

The current study found that African American, Hispanic, and other racial and ethnic minority adults experienced relatively improved outcomes in states that adopted Medicaid expansion after ACA implementation. The major amputation rates for racial and ethnic minority adults with DFUs significantly blunted in early-adopter states compared with nonadopter states after Medicaid expansion. This finding could be because of the recruitment of uninsured adults into the Medicaid program. Importantly, Medicaid beneficiaries in early-adopter states did not experience a significant change in the amputation rate despite the transition of at-risk uninsured adults into the Medicaid program. Taken together, this study highlights the potential benefit of the ACA for populations at disproportionately elevated risks of diabetes-related amputation.
